# Designing questionnaires: healthcare survey to compare two different response scales

**DOI:** 10.1186/1471-2288-14-96

**Published:** 2014-08-03

**Authors:** Salome Dell-Kuster, Esteban Sanjuan, Atanas Todorov, Heidemarie Weber, Michael Heberer, Rachel Rosenthal

**Affiliations:** 1 Basel Institute for Clinical Epidemiology and Biostatistics, University Hospital Basel, Hebelstrasse 10, 4031 Basel, Switzerland; 2 Department of Surgery, University Hospital Basel, 4031 Basel, Switzerland; 3 Department of Medical Processes and Quality, Basel University Hospital, 4031 Basel, Switzerland

**Keywords:** Quality assurance, Patient satisfaction, Questionnaire, Answering scale

## Abstract

**Background:**

A widely discussed design issue in patient satisfaction questionnaires is the optimal length and labelling of the answering scale. The aim of the present study was to compare intra-individually the answers on two response scales to five general questions evaluating patients’ perception of hospital care.

**Methods:**

Between November 2011 and January 2012, all in-hospital patients at a Swiss University Hospital received a patient satisfaction questionnaire on an adjectival scale with three to four labelled categories (LS) and five redundant questions displayed on an 11-point end-anchored numeric scale (NS). The scales were compared concerning ceiling effect, internal consistency (Cronbach’s alpha), individual item answers (Spearman’s rank correlation), and concerning overall satisfaction by calculating an overall percentage score (sum of all answers related to the maximum possible sum).

**Results:**

The response rate was 41% (2957/7158), of which 2400 (81%) completely filled out all questions. Baseline characteristics of the responders and non-responders were similar. Floor and ceiling effect were high on both response scales, but more pronounced on the LS than on the NS. Cronbach’s alpha was higher on the NS than on the LS. There was a strong individual item correlation between both answering scales in questions regarding the intent to return, quality of treatment and the judgement whether the patient was treated with respect and dignity, but a lower correlation concerning satisfactory information transfer by physicians or nurses, where only three categories were available in the LS. The overall percentage score showed a comparable distribution, but with a wider spread of lower satisfaction in the NS.

**Conclusions:**

Since the longer scale did not substantially reduce the ceiling effect, the type of questions rather than the type of answering scale could be addressed with a focus on specific questions about concrete situations instead of general questions. Moreover, the low correlation in questions about information provision suggests that only three possible response choices are insufficient. Further investigations are needed to find a more sensitive scale discriminating high-end ratings. Otherwise, a longitudinal within-hospital or a cross-sectional between-hospital comparison of patient care is questionable.

## Background

Measuring patients’ perception of hospital care after in-patient treatment is an important tool to identify strengths, weaknesses and unmet needs in healthcare services, as well as to identify changes in patient satisfaction over time. The ultimate goal is to improve the quality of healthcare. Moreover, it allows the comparison of different hospitals’ performance. Patients’ perception of hospital care is influenced by many different factors. Typical determinants of healthcare satisfaction are humaneness and informativeness of communication with the patient, as well as the overall medical quality and competence [1-3].

Patient satisfaction is frequently assessed using questionnaires. When designing such questionnaires, several aspects need to be carefully evaluated in order to achieve a reliable and valid instrument. A prominent controversy about questionnaires is the optimum number of response categories. Jacoby and Matell [4] proclaim that a three-point Likert scale (bipolar scale) [5] provides an appropriate discrimination and validity. On the other hand, Preston and Colman [6] could show that reliability, validity and discrimination were significantly higher in scales with up to about seven response categories. Moreover, respondents considered scales with up to seven response categories a good compromise between ease of use and discriminative capacity.

An additional issue of debate when designing questionnaires is whether all scale points, or only the end points, should be labelled with descriptors. Garratt and colleagues compared a 5-point scale with descriptors for all scale points to a 10-point scale with end-point descriptors only [7]. In contrast to the highly skewed distributions for the 10-point scale, they found quite symmetric distributions with low floor and ceiling effects for the five-point scale. A ceiling effect occurs when a measure possesses a distinct upper limit for potential responses and a large number of patients score at or near this limit (the opposite of a floor effect). A high ceiling effect renders discrimination at the high-end difficult and reduces the possibility of measuring further improvement. Furthermore, it deteriorates the validity and reliability of the findings [8] by hampering the estimation of the central tendency and variance of the data. Apart from the length of the response scale, several other methods have been proposed in the literature to reduce ceiling effects. One example is to change the response form from an evaluating to a reporting style [9], or alternatively to focus on the content and structure of the questions themselves instead of the type of answering scale, such as by asking concrete questions about relevant situations during the hospital stay, or by applying a multi-item technique [10,11].

At our institution, the permanent patient questionnaire consists of 17 reporting questions on concrete relevant situations applying a multi-item technique. Its response scale is displayed on a labelled adjectival scale with three to four categories. Additionally, our institution annually sends out five questions within the framework of the Swiss National Quality Contract (ANQ) for nationwide benchmarking purposes during a limited period of time. This minimal set of general questions about important determinants of patient satisfaction has been defined both to enable and to stimulate the additional inclusion of more extended and hospital-specific questions. The response scale is displayed using an 11-point numeric scale (NS) with anchors at both ends. These five questions are redundant to five questions out of the 17-item institutional questionnaire.

In the present study, we used this setting of two different response scales of the same questions for an intra-individual comparison between the answers of these redundant questions. Specifically, we address the influence of the answering scale on the ceiling effect and the internal consistency and we compare the sets of responses on an individual item as well as on an overall level. We anticipated that the internal consistency is higher and the ceiling effect less pronounced using a longer response scale, leading to a better discrimination in high ratings.

## Methods

### Participants and setting

From November 2011 until January 2012, every in-hospital patient above 16 years of age who was discharged from the University Hospital in Basel, Switzerland, had the opportunity to participate in this healthcare survey. Patients received a letter with both questionnaires and were requested to accept the redundancy of five questions on two different response scales and to answer all questions. If the discharged patients did not return the questionnaire, no reminders were sent out. The questionnaire was sent out in the following five languages: German, French, Italian, English and Turkish. If a patient had a different native language, they received the German version of the questionnaire.

The following socio-demographic variables were assessed for all patients: Age, gender, nationality, native language, emergency versus elective hospital admission, length of hospital stay, individual department where patients received their main treatment and hospital readmissions within the same study period. Since our anonymisation procedure was irreversible, re-admission information was limited to all in-hospital patients during the study period and was not available for the subpopulation of patients returning the complete questionnaire.

The anonymisation procedure of this quality control survey was approved by the local data protection committee (“Datenschutzbeauftragter des Kantons Basel-Stadt”, reference number 10_0147). Since the primary purpose of the survey was the quality control of in-hospital patients at the University Hospital Basel, the local ethics committee (“Ethikkommission beider Basel”) exempted the survey from formal ethical committee approval. Moreover, confidentiality was preserved by separating the data analysis team from the healthcare providers.

### Questionnaires

#### 1) Questionnaire using a numeric answering scale (NS)

In 2011, the Swiss National Association for Quality Development in Hospitals and Clinics (ANQ, http://www.anq.ch) has developed a questionnaire with the aim that every member of this association uses the questionnaire during one defined month of the year, leading to a yearly national quality evaluation of all hospitals in Switzerland. In order to obtain a larger sample, we have extended the survey to three months.

**Figure 1:**
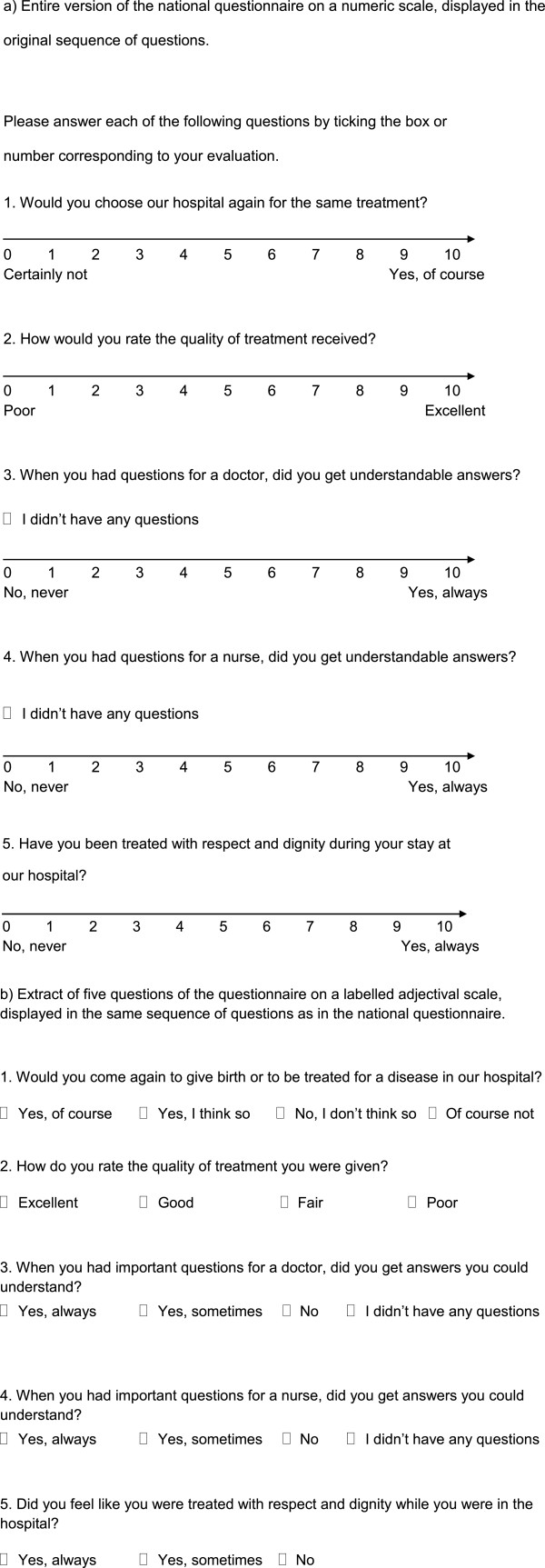
Questionnaire on a numeric (NS) and on a labelled adjectival (LS) response scale.

The five questions (Figure 1) covered four important domains, which indirectly rate patient satisfaction on an evaluation scale. The questions were ordered as follows: 1) behavioural intent to return to the hospital (one question), 2) quality of treatment (one question), 3) quality of medical information (two questions), and 4) question concerning judgement whether the patient was treated with respect and dignity (one question). The response scale is displayed on an 11-point numeric scale (NS) with anchors at both ends, presenting the negative answers first.

#### 2) Questionnaire using a labelled answering scale (LS)

The questionnaire consists of 17 items (Figure 1 and webappendix: Additional file 1), whereby for the present study only the five questions, which are shared with the national survey, were considered. The questionnaire started with questions about the quality of information, followed by the question about respect and dignity, the quality of treatment and ended with the question about the intent to return. This questionnaire is based on the Picker questionnaire, which is a widely used and validated instrument for quality evaluation in hospital contexts [12]. The response scale is displayed on an adjectival scale with, according to the question, three or four labelled response categories, presenting the positive answers first. The labels included the following answering options: 1) Yes, of course; yes, I think so; no, I do not think so; of course not and 2) excellent; good; fair and poor; and 3) with three response categories yes, always; yes, sometimes; no.

### Statistical analyses

Statistical analyses were conducted using Intercooled Stata Version 11.2 for Macintosh (StataCorp, College Station, TX, USA). Graphs were performed in Intercooled Stata Version 11.2 for Macintosh and in R system, version 2.14.2 (R Foundation for Statistical Computing. Vienna, Austria). We report 95% confidence intervals (CI), rather than p-values, in order to emphasise clinical relevance over statistical significance, because in this large data set, irrelevant differences are also statistically significant. According to the current guidelines for reporting observational data (STROBE) [13], we avoided significance tests for the evaluation of differences in baseline characteristics.

Baseline characteristics were summarised for all patients, questionnaire responders, non-responders and responders with no missing items. The latter group corresponds to the patients included in this analysis.

The answers on both response scales were summarised in a frequency table for comparison of the ceiling effect. The Cronbach’s alpha for the five questions on each response scale was calculated as a measure for internal consistency. Since the five items are all measuring patient satisfaction (i.e. are congeneric), the alpha is an estimate of the lower end of reliability [14]. Therefore, only the lower one-sided 95% confidence interval was computed to see whether it is significantly greater than some minimal value [15,16]. Furthermore, the data were investigated using graphical displays (histogram, scatterplot) and the Spearman’s rank correlation coefficient with its 95% confidence intervals (CI) [17] to determine the amount of correlation between both response scales (NS and LS) for each item.

We calculated an overall percentage score for each of the two questionnaires’ results as a summary measure with a common metric. In the numeric scale questionnaire, we calculated the sum of the five answers, ranging from 5 to 55. In the adjectival scale questionnaire, we attributed values from 1 to 4 or 1 to 3, respectively, to the answer categories, resulting in a range from 5 to 17. Here, we assumed equal distance between the categories. The percentage score was calculated by dividing the sum of the answers by the maximum possible sum. For the calculation of the Spearman’s correlation coefficient and the overall percentage score, those patients who did not have any questions in the third and fourth questions were disregarded.

These analyses were repeated in an explorative way in the following prespecified subgroups: Elective versus emergency patients and short versus long hospitalisation, as defined by median split. We have chosen these subgroups since it is known from the literature that hospital length of stay and type of admission have an impact on satisfaction with hospital care [18,19].

## Results

### Patients’ characteristics

**Figure 2:**
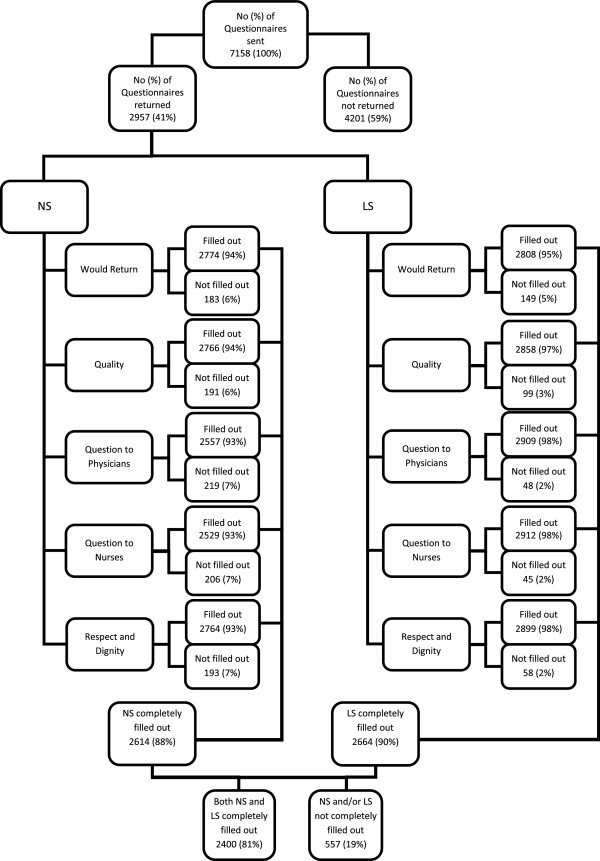
**Flow chart.** Flow chart of the numbers of questionnaires sent, the number of questionnaires returned and the number of individual questions answered. Baseline characteristics were calculated i) for patients who returned the questionnaire, ii) for patients who did not return the questionnaire and iii) for patients who returned the questionnaire and who completely filled out all questions. The latter correspond to the patients included in the analysis comparing both response scales. NS = Numeric Scale, LS = Labelled Scale. NA refers to not applicable as answering option in the two questions about comprehensibility of the answers from physicians and nurses. In this case NA means the patient did not have had any questions.

Of the 7158 patients to whom the questionnaire was sent, 2957 (41%) responded. Out of these, 2400 (81%) completely filled out all questions on both response scales (Figure 2). The number of all patients who stayed more than once in the hospital during the study period was 603 (8.3%).

In responders versus non-responders, the percentage of patients after elective hospitalisation was higher (63% versus (vs.) 51%) and the percentage of Swiss nationality was slightly higher (76% vs. 69%). The distribution regarding age, gender and native language was comparable (Webappendix: Additional file 2). Patients who completely filled out all questions (Webappendix: Additional file 3) showed similar baseline characteristics as those who sent back the questionnaire (Table 1).

**Table 1:** Baseline characteristics of patients with returned questionnaire

	**Total (n = 2957)**	**Length of hospital stay (n = 2957)**		**Admission (n = 2934)**	
		**Length of stay ≤ 4 days, n = 1482 (50%)**	**Length of stay > 4 days, n = 1475 (50%)**	**Emergency admission, n = 1072 (37%)**	**Elective admission, n = 1862 (63%)**
**Age in years**, mean (SD)	60 (19)	56 (19)	63 (18)	64 (19)	57 (18)
**Gender**, n (%)					
Male	1419 (48%)	701 (47%)	718 (49%)	566 (53%)	839 (45%)
Female	1538 (52%)	781 (53%)	757 (51%)	506 (47%)	1023 (55%)
**Length of hospital stay**, median (IQR)	4 (2 – 9)	2 (1–3)	9 (7 – 13)	6 (2 – 11)	4 (2–8)
**Hospitalisation,** n (%)					
Emergency	1072 (36%)	435 (29%)	637 (43%)		
Elective	1862 (63%)	1042 (70%)	820 (56%)		
Not defined	23 (1%)	5 (<1%)	18 (1%)		
Length of Stay ≤ 4 Days	1482 (50%)			435 (41%)	1042 (56%)
Length of Stay > 4 Days	1475 (50%)			637 (59%)	820 (44%)
**Department**, n (%)					
Surgery	1178 (40%)	422 (28%)	756 (51%)	413 (39%)	744 (40%)
Internal Medicine	943 (32%)	498 (34%)	445 (30%)	517 (48%)	426 (23%)
Gynaecology and Obstetrics	442 (15%)	281 (19%)	161 (11%)	26 (2%)	415 (22%)
Otorhinolaryngology	109 (4%)	91 (6%)	18 (1%)	15 (1%)	93 (5%)
Radiology	80 (3%)	80 (5%)	0 (0%)	0 (0%)	80 (4%)
Ophthalmology	64 (2%)	59 (4%)	5 (<1%)	12 (1%)	52 (3%)
Geriatric Medicine	58 (2%)	2 (<1%)	56 (4%)	52 (5%)	6 (<1%)
Intensive Care Unit	46 (2%)	42 (3%)	4 (<1%)	31 (3%)	15 (1%)
Dermatology and Venereology	37 (1%)	7 (<1%)	30 (2%)	6 (1%)	31 (2%)
**Nationality,** n (%)					
Swiss	2243 (76%)	1067 (72%)	1176 (80%)	854 (80%)	1373 (74%)
German, Austrian, Liechtensteiner	224 (8%)	123 (8%)	101 (7%)	63 (6%)	159 (9%)
French	35 (1%)	22 (1%)	13 (1%)	7 (1%)	28 (2%)
Italian	99 (3%)	48 (3%)	51 (3%)	42 (4%)	56 (3%)
English, Irish	11 (<1%)	5 (<1%)	6 (<1%)	7 (1%)	4 (<1%)
Turkish	61 (2%)	36 (2%)	25 (2%)	18 (2%)	42 (2%)
European, other	170 (6%)	105 (7%)	65 (4%)	54 (5%)	115 (6%)
US-American, Canadian, Australian	18 (1%)	17 (1%)	1 (<1%)	4 (<1%)	13 (1%)
Extra-European, other	55 (2%)	34 (2%)	21 (1%)	20 (2%)	35 (2%)
Missing	41 (1%)	25 (2%)	16 (1%)	3 (<1%)	37 (2%)
**Language**, n (%)					
German	2793 (94%)	1382 (93%)	1411 (96%)	1024 (96%)	1749 (94%)
French	45 (2%)	27 (2%)	18 (1%)	9 (1%)	36 (2%)
Italian	37 (1%)	19 (1%)	18 (1%)	19 (2%)	16 (1%)
English	30 (1%)	24 (2%)	6 (<1%)	11 (1%)	19 (1%)
Turkish	25 (1%)	14 (1%)	11 (1%)	4 (<1%)	20 (1%)
Others	27 (1%)	16 (1%)	11 (1%)	5 (<1%)	22 (1%)

Characteristics of patients who returned their questionnaire: all patients (n = 2957 (41%)) and within the pre-defined subgroups short versus long hospital stay (n = 1482 (50%) vs. n = 1475 (50%)) and emergency versus elective admission (n = 1072 (37%) vs. n = 1862 (63%)).

### Response scale comparison

#### 1) Questionnaire results, mapping and ceiling effect

**Figure 3:**
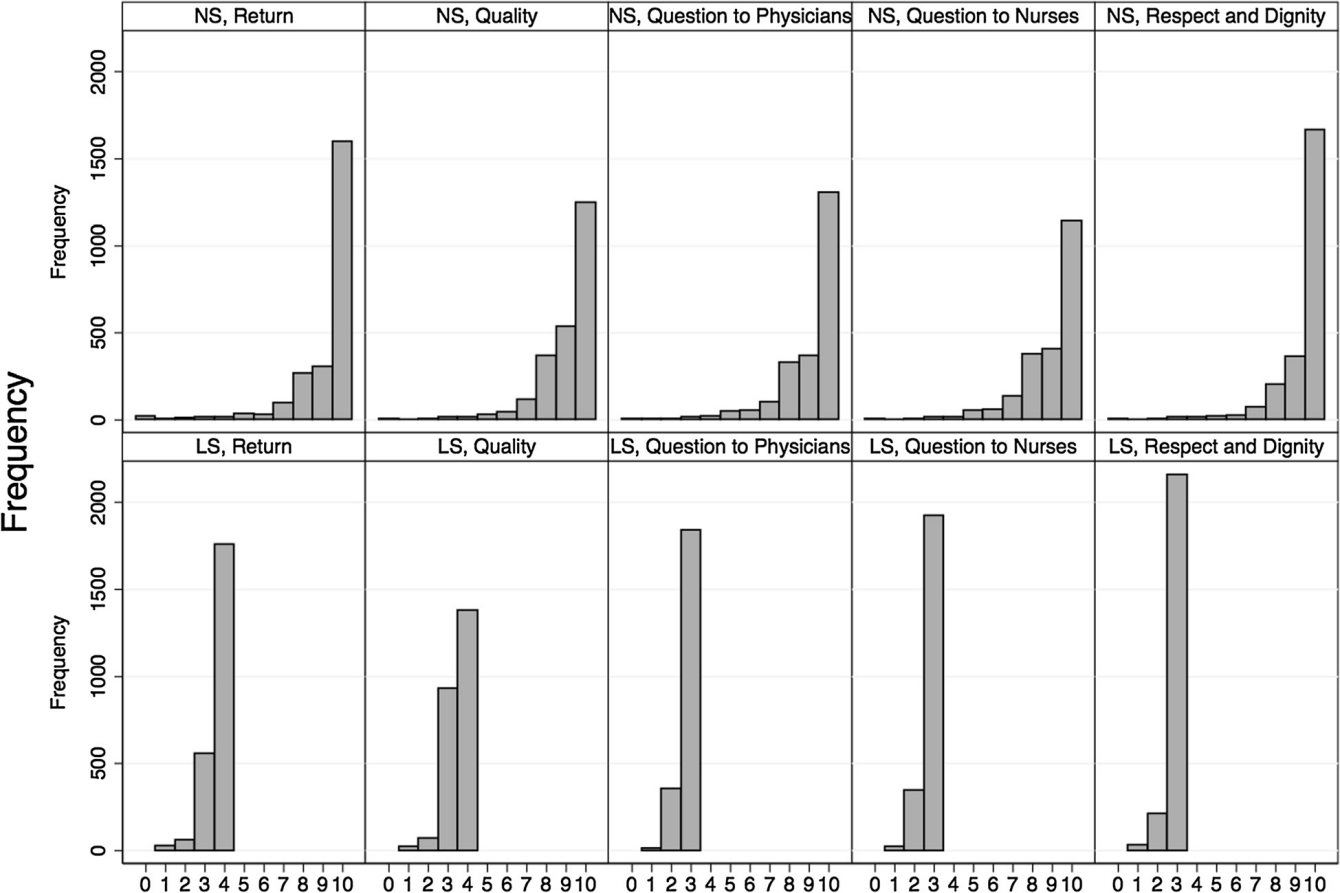
**Questionnaire results on the numeric and labelled response scale.** Frequency distribution of the answers to each item on both scales separately. NS = Numeric Scale, LS = Labelled Scale.

**Figure 4:**
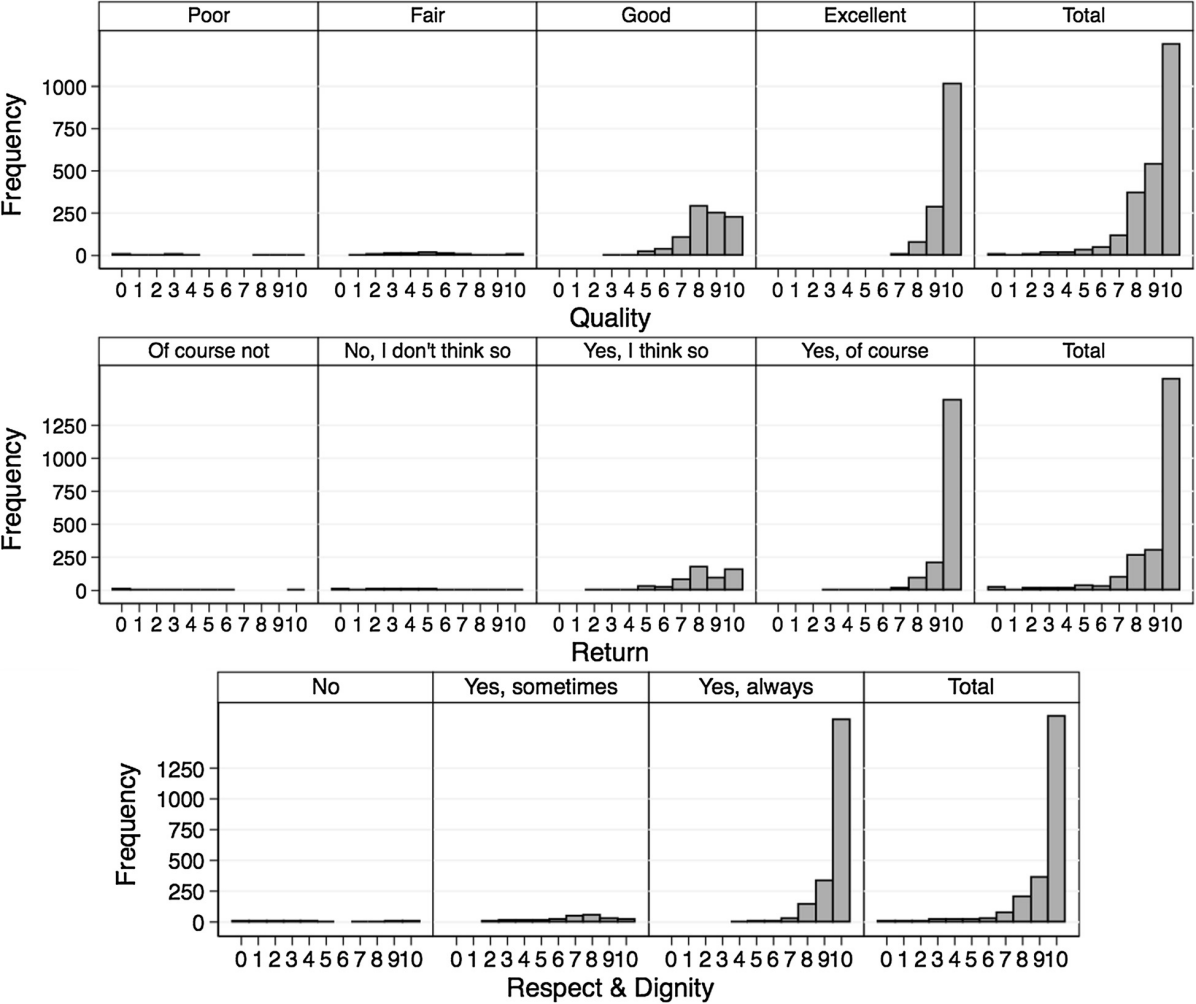
Mapping of the answers of the patients on both scales - Questions quality of treatment, behavioural intent to return and judgement whether treated with respect and dignity.

**Figure 5:**
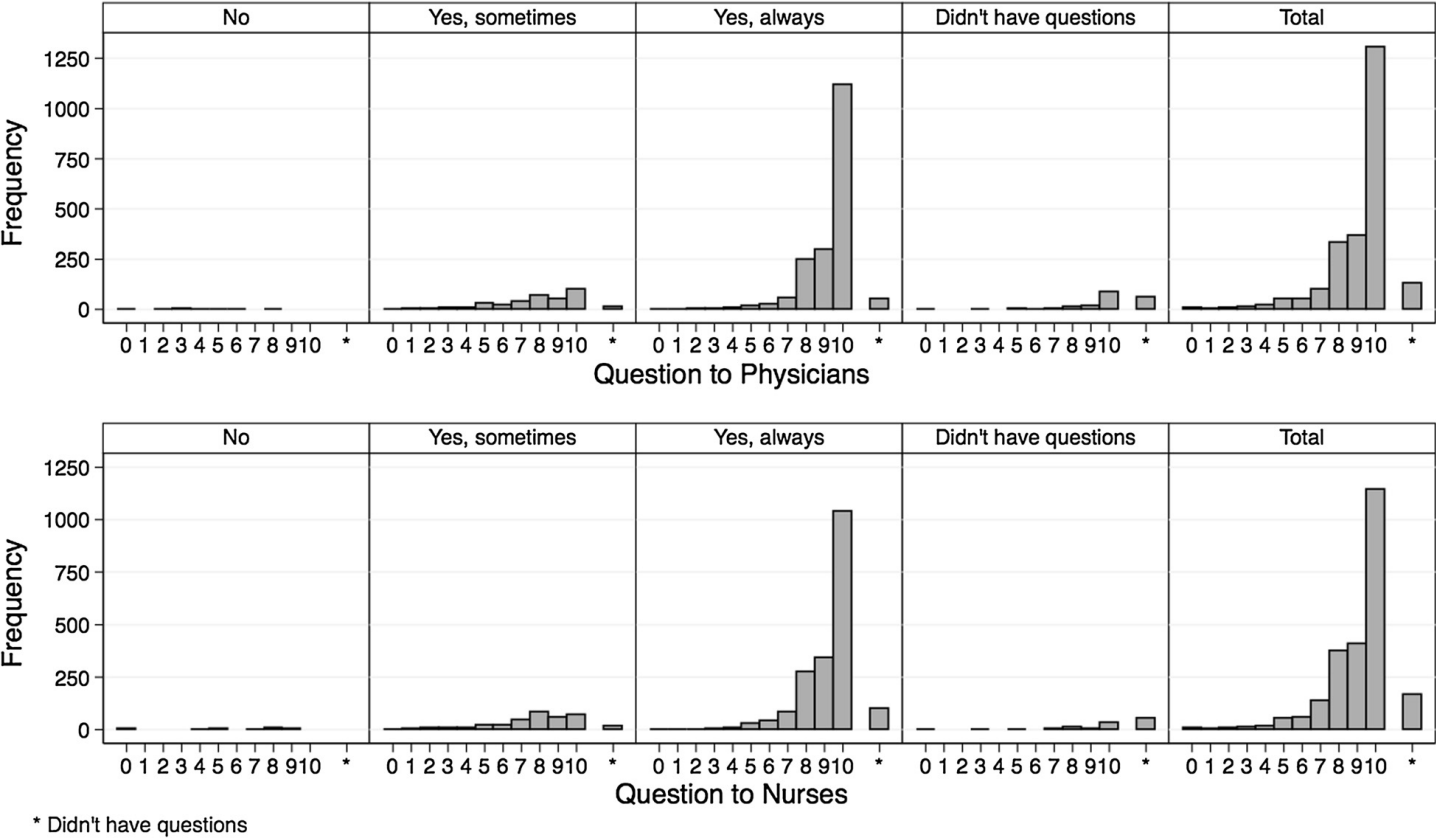
Mapping of the answers of the patients on both scales - Questions quality of medical information by physicians and by nurses.

Summary statistics for the answers on the NS and the LS show a very high and satisfactory rating in all domains (Table 2 and Figure 3). As shown in the mapping of the answers of each category of the LS to the NS (Figures 4 and 5), each response category of the adjectival response scale is depicted by at least four levels on the numeric response scale. Comparing both response scales, the percentages of best category ratings in the LS are slightly higher for questions with four answering categories as compared to the NS (e.g. 73% in LS vs. 67% in NS for the “Return-Question”). This ceiling effect was clearly more pronounced in the LS with only three answering categories (e.g. 80% on LS vs. 48% on NS for “Question to Nurses”). The floor effect was more pronounced in the LS with the lowest two to four categories of the NS being represented by the lowest category of the LS, independently of the number of answering categories in the LS.

**Table 2:** Questionnaire results on a numeric or a labelled adjectival response scale (n = 2400)

	**Numeric Scale (NS)**												**Labelled Scale (LS)**				
**Questions**	**0**	**1**	**2**	**3**	**4**	**5**	**6**	**7**	**8**	**9**	**10**	**NA**	**A**	**B**	**C**	**D**	**NA**
Would Return	1%	<1%	1%	1%	1%	2%	1%	4%	11%	13%	67%	-	1%	2%	23%	73%	-
Quality	<1%	<1%	<1%	1%	1%	1%	2%	5%	15%	22%	52%	-	1%	3%	39%	57%	-
Question to Physicians	<1%	<1%	<1%	1%	1%	2%	2%	4%	14%	15%	54%	6%	1%	15%	77%	-	8%
Question to Nurses	<1%	<1%	<1%	1%	1%	2%	3%	6%	16%	17%	48%	7%	1%	14%	80%	-	5%
Respect and Dignity	<1%	<1%	<1%	1%	1%	1%	1%	3%	8%	15%	69%	-	1%	9%	90%	-	-

NA = I didn’t have any questions for staff.

The answering scale in the NS had anchoring labels at 0 and 10 of ‘Certainly not – Yes, of course’ for the first question, ‘Poor – Excellent’ for the second question, and ‘No, never – Yes, always’ for questions 3 to 5, respectively.

The categories of the answering scale in the LS were labelled with ‘Of course not (A), No, I don’t think so (B), Yes, I think so (C), Yes, of course (D)’ for the first question; ‘Poor (A), Fair (B), Good (C), Excellent (D)’ for the second question; and ‘No (A), Yes, sometimes (B), Yes, always (C) for questions 3 to 5, respectively.

#### 2) Comparison of internal consistency of each response scale

Cronbach’s alpha for all five questions was 0.77 on the LS with a lower bound of the confidence interval of 0.755 as opposed to 0.89 on the NS with a lower bound of the confidence interval of 0.886.

#### 3) Response scale comparison on the individual item level

**Figure 6:**
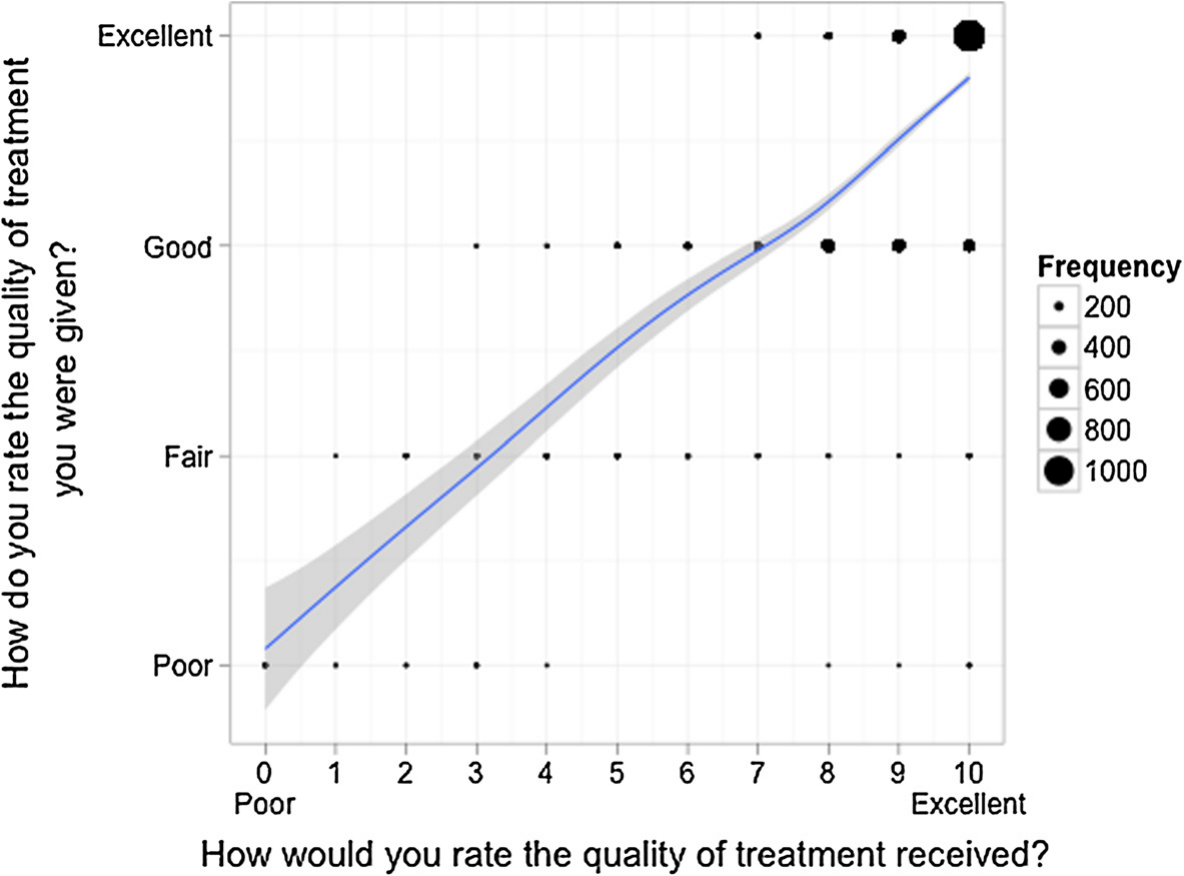
**Correlation of the answers of patients on both scales - Question quality of treatment.** Scatterplot for comparison of the answers of each patient on two different answering scales with an overlaid smoother for each item separately. The dots are proportional to the frequency of the corresponding combination.

**Figure 7:**
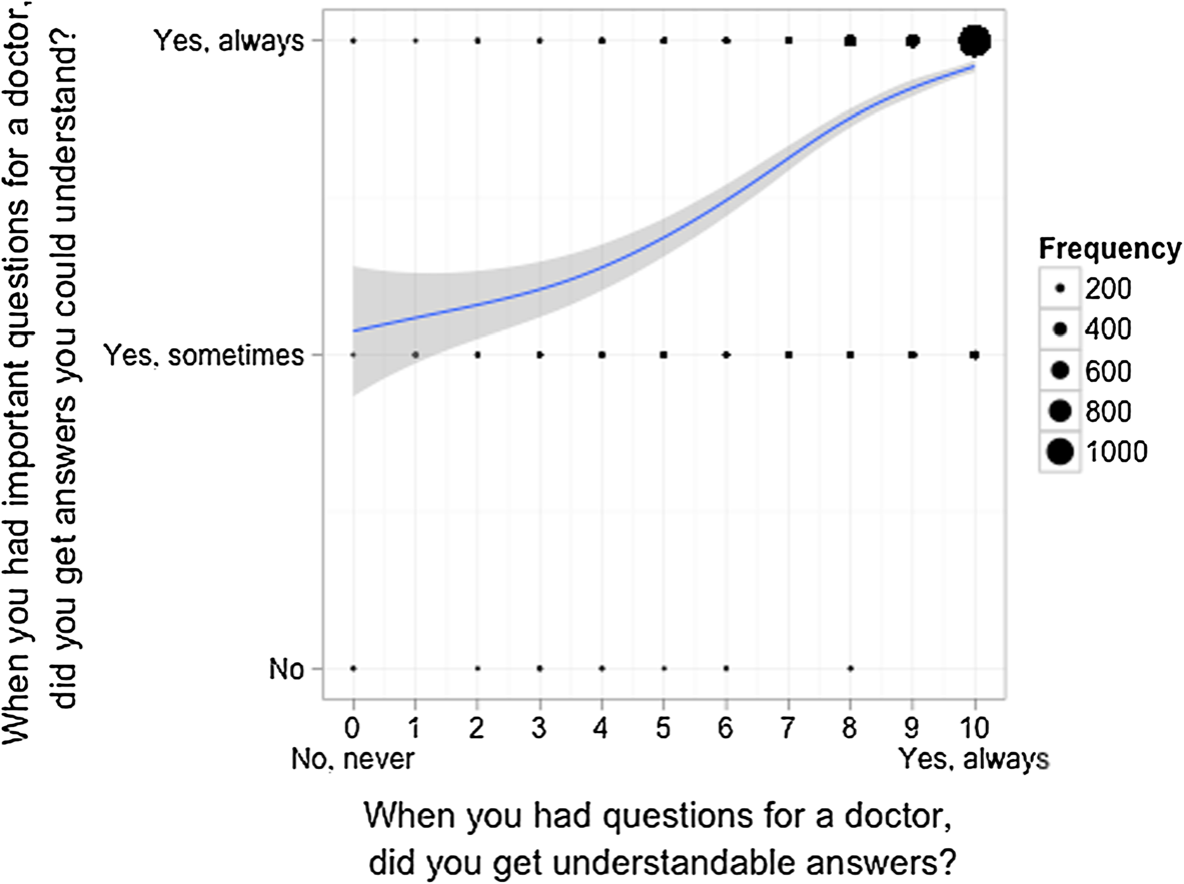
**Correlation of the answers of patients on both scales - Question quality of medical information by physicians.** Scatterplot for comparison of the answers of each patient on two different answering scales with an overlaid smoother for each item separately. The dots are proportional to the frequency of the corresponding combination.

The results of the correlation of the individual item answers between both response scales are displayed in Table 3 (all questions) and Figures 6 and 7 for two representative questions (quality of treatment and quality of information provided by physicians; Webappendix: Additional files 4, 5 and 6 for the remaining three questions). There was a strong correlation between the response scales regarding questions about intent to return, quality of treatment and judgement whether the patient was treated with respect and dignity, but a lower correlation concerning satisfactory information transfer by physicians or nurses.

**Table 3:** Spearman’s rank correlation coefficient between the NS and the LS for each item

	**All patients (n = 2400)**	
**Question**	**Correlation coefficients**	**95% confidence intervals**
Return	0.60	0.58, 0.63
Quality	0.59	0.56, 0.61
Question to Doctors	0.32	0.29, 0.36
Question to Nurses	0.33	0.29, 0.37
Respect and Dignity	0.52	0.49, 0.55

Spearman’s rank correlation coefficient for each item between both response scales (NS and LS): all patients (n = 2400 for question 1, 2, and 5, n = 2140 for question 3 and n = 2173 for question 4, respectively).

Corr. Coeff. Correlation Coefficient. CI Confidence Interval.

#### 4) Response scale comparison on an overall level using a percentage score

**Figure 8:**
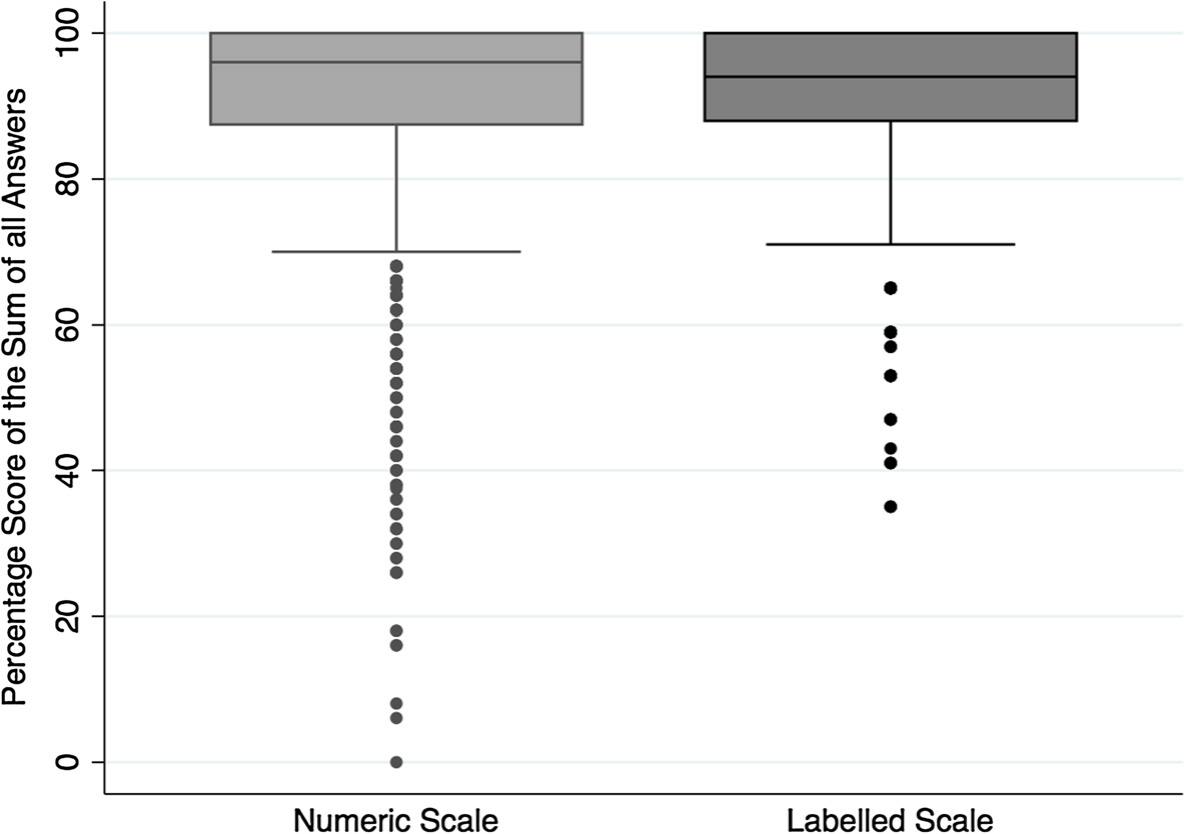
Percentage score of the total sum of answers on the NS and LS.

**Figure 9:**
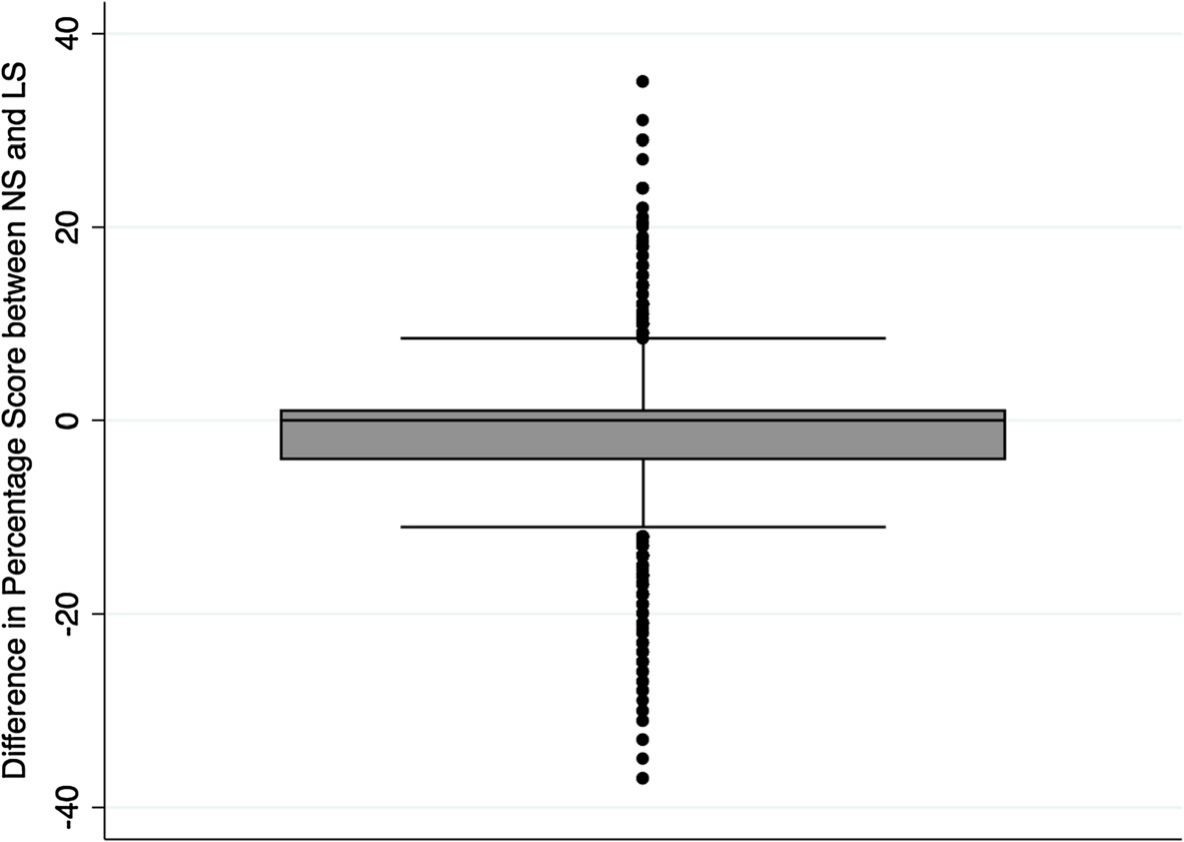
Differences of percentage score between the NS and LS.

The overall percentage score showed a comparable distribution (median 96 (interquartile range (IQR) 87.5 – 100) for the NS vs. 94 (IQR 88 – 100) for the LS, respectively) with a wider spread of lower satisfaction in the NS (Figures 8 and 9).

### Response scale comparison in pre-specified subgroups

Patient satisfaction was rated higher in patients after elective, than after emergency admission and after shorter, compared to longer, hospital stay. Consequently, the ceiling effect was more pronounced on both response scales in patients after elective admission and shorter stay (Table 2). The results of the comparison of Cronbach’s alpha, the individual items and of the overall percentage score did not greatly differ from overall results (Webappendix: Additional file 7).

## Discussion

This intra-individual answering scale comparison shows that patients’ perception of hospital care in this public teaching hospital is high without a substantial reduction in floor and ceiling effects on the numeric compared to the labelled adjectival response scale. Moreover, the overlap of the numerical, when plotted against the categorical response scale indicates the difficulty of defining patient groups with regard to their satisfaction with healthcare. The Cronbach’s alpha is clearly higher using the numeric response scale, whereas the individual item level correlation between the response scales is high in questions about intent to return, quality of treatment and patient care was performed with respect and dignity, but low regarding satisfactory information transfer. Finally, the distribution of the percentage score is comparable on both response scales, which leads to a high overall correlation.

### Strengths of the study

Our study is one of the largest studies including all departments of a university hospital. It belongs to a small number of studies, using a pragmatic (as opposed to an empirical) approach [3]. Moreover, this study is unique since it enables a within-patient comparison of the perception of hospital care. This allows refining the properties of the response scale in the general questions in order to reduce floor and ceiling effect which in turn leads to a better discrimination at the high end rating and to improved internal consistency.

### Limitations of the study

Our study presents some limitations. The main limitation was that only overall questions on patient satisfaction were available for the response scale comparison, since the nationwide questionnaire was limited to these five evaluating questions. A further limitation was a response rate of only 41%, which however is in line with response rates between 42 and 48% in previous studies [7,20]. Resulting bias is unlikely since the baseline characteristics of responders and non-responders only differed slightly and the percentage of missing items was very small compared to other studies in similar settings [21,22] still allowing for a certain generalisability. Moreover, several studies could show that non-respondents are generally not significantly different from respondents in terms of satisfaction scores [23], or in terms of sociodemographic characteristics [24]. A further limitation consists of differences between the questions on both response scales with regard to exact wording, the polarity of the response scale, the order of the questions and that the five questions on the LS correspond to a subset of a 17-items questionnaire, whereas the nationwide survey only included these five general questions. This may partly explain the divergent answers on both answering scales, which results in lower correlation coefficients and could be taken as a consequence of the pragmatic approach.

### Findings in relation to other studies

The high patient satisfaction in the present study is in line with the findings of an international evaluation [25], in which Switzerland was found to stand out by having very high quality of care ratings compared to other European countries and the United States.

Questionnaires are convenient for monitoring in-patient satisfaction, but reliability and validity of the findings are reduced by skewed response distributions [8]. Different methods have been proposed to reduce the ceiling effect. In our study, we investigated the influence of the length of scale together with labelled categories versus end-anchors. We found a less pronounced ceiling effect with a persistent albeit less left-skewed response distribution on the longer end-anchored numeric scale than on the shorter adjectival scale with labelled categories. It was rather unexpected that the skewedness on the numeric answering scale (compared to LS) was nearly as marked. The different polarity of both answering scales, with the LS having positive answers on the left-hand side and the NS on the right-hand side, might partially explain the higher ceiling effect found in the answers on the LS, since there is evidence of bias towards the left side [26]. People tend to use the first satisfactory response option in a presented questionnaire, as shown in studies which directly compared a reversed to a not-reversed answering scale [27,28]. However, had the reversed answering scale of both questionnaires a strong influence in our setting, we would expect the response distribution of the numeric scale (with negative answers on the left-hand side) to be more symmetrical. Moret et al. [20] achieved a reduction in ceiling effect by extending the response scale from a four-point to a five-point format, but with an unbalanced scale including three positive and two negative choices. In contrast to our results, Garratt et al. [7] showed that a 10-point scale produced a highly skewed distribution compared to a balanced 5-point scale, which showed a fairly symmetrical distribution. However in both cited studies, the ceiling effect was less pronounced than in our data [7,20]. Similarly, in a randomised comparison of four patient satisfaction questionnaires, Perneger et al. [29] found the lowest ceiling effect in a questionnaire using an unbalanced five-point Likert scale. These results suggest that an unbalanced five or six points scale might outperform both our three- to four-point scale as well as our 11-point end-anchored scale. Alternatively, an even stronger imbalance with four positive categories is necessary to render our high rating approximately normally distributed. A normal distribution of questionnaire results can also be achieved by using an overall score [30,31]. However, even by combining all five answers to an overall percentage score, we did not obtain a fairly symmetric distribution. It might be argued that it is not possible to achieve a symmetrical distribution with general questions about patient satisfaction [32,33], rendering the selection of the questions used for the national benchmark disputable.

Apart from changing the length of the answering scale, the two scales also differed in the number of labelled categories. The use of an adjectival scale for comparative purposes can be limited by its lack of sensitivity for detecting small changes and may strongly depend upon the choice of the wording and the literacy of the patient. Accordingly, Downie et al. showed an improvement of discrimination by using a numerical rating scale as compared to a four-point descriptive scale and a continuous scale with two end-anchors [34]. On the other hand, Streiner and Norman provided evidence that there is a tendency of end-labelled scales to pull responses to the end [35]. This is in line with our data where a fairly big difference could be found between the percentages in the highest category (10) and the next one (9) in the responses on the numerical scale using end-anchors. Garratt et al. argued that the higher ceiling effect of their longer 10-point scale could be explained by this phenomenon of end-anchors pulling responses to the end [7]. On the other hand, one could expect an end-aversion bias, especially if the endpoints are labelled with absolute words such as ‘Always’ as opposed to ‘Almost always’.

Our results further suggest that the internal consistency (Cronbach’s alpha) was higher for the NS than for the LS, which has been confirmed in the study of Hendriks et al. [21]. However, with a lower limit of the Cronbach’s alpha above 0.75, both scales can formally be accepted as a valid measure of patient satisfaction.

Comparing both answering scales, we found a high individual item level correlation in three out of five questions. These three questions cover the domains of intention to return, quality of treatment and respectful behaviour. Laerhoven et al. [36] found Spearman rank correlation coefficients comparing three different types of response scales, which all laid above the plausible range of values from our study. This finding might be explained by the above-mentioned additional differences in both questionnaires apart from the length and type of scale (e.g. polarisation and order of questions). There is consistent evidence that the most important factor affecting patient satisfaction is the patient-practitioner relationship, including information provision [2,3]. In the present study, this was the domain in which the correlation between both response scales diverged most. From the collected information, we are unable to determine which of the questionnaires contains the valid individual response. On the other hand, it is not unexpected that the correlation is lower in these two information questions. Whereas questions about treatment with respect and dignity and intention to return imply almost binary answers, in the sense that you are either willing to return or not, a question about provision with adequate information is much more complex, depending on the content as well as the wording of the given information. Moreover, the adjectival response scale only consists of three categories not allowing for much differentiation.

### Implications for daily practice and further research

In order to further improve the nationwide benchmark at this point, the questions and answering scale need to be refined to enhance the discriminative power for the high end of patient satisfaction, since both response scales showed a strong ceiling effect. This is especially relevant, because patient satisfaction may influence many important outcomes such as compliance, overall well-being and consumer choice. The choice of these five overall questions for a national benchmark should be challenged and a different set of reporting questions defining quality aims tested.

## Conclusions

The longer numeric scale did not substantially reduce the ceiling effect in these five general questions. This is to some extent due to a very high patient satisfaction rating in our data. To increase the discriminative power at the high-end of patient satisfaction, the answering scale needs to be refined, for instance by the use of an unbalanced scale. Additionally, the content of the questions could be changed from general to specific questions reporting about concrete situations known to have a high impact on patient satisfaction. The ultimate goal is to provide high-quality medical care with excellent patient satisfaction and to be able to have an optimal tool for longitudinal and cross-sectional comparisons.

## Abbreviations

NS: Numeric scale; LS: Labelled scale; NA: Not applicable; Vs: Versus; IQR: Interquartile range; CI: Confidence interval.

## Competing interests

The authors declare that they have no competing interests.

## Authors’ contributions

SDK participated in the design of the study, performed the statistical analysis and drafted the manuscript. ES participated in the design of the study, performed part of the statistical analysis and critically revised the manuscript. AT participated in the design of the study, coordinated data collection and critically revised the manuscript. HW and MH participated in the design of the study, in the interpretation of the results and critically revised the manuscript. RR participated in the design of the study, the interpretation of the results, and helped to draft the manuscript. All authors read and approved the final manuscript.

## Pre-publication history

The pre-publication history for this paper can be accessed here:

http://www.biomedcentral.com/1471-2288/14/96/prepub

## Supplementary Material

Additional file 1 **Questionnaire on labelled response scale, entire version.** Entire version of the questionnaire on a labelled adjectival response scale, displayed in the original sequence of questions. Questions number 1, 2, 7, 16 and 17 correspond to the questions 3, 4, 5, 2 and 1 respectively of the national survey. Click here for file

Additional file 2 **Baseline characteristics of patients without returned questionnaire.** Characteristics of patients who did not their questionnaire: All patients (n = 4201 (59%)) and within the pre-defined subgroups short versus long hospitalisation (n = 2243 (53%) vs. n = 1958 (47%)) and emergency versus elective hospitalisation (n = 2040 (49%) vs. n = 2122 (51%)). Click here for file

Additional file 3 **Baseline characteristics of patients with entire completion of all questions on both scales.** Characteristics of patients who returned the questionnaire and completely filled out all questions on both response scales corresponding to the analysis population for response scale comparison: All patients (n = 2400 (34%)) and within the pre-defined subgroups short versus long hospitalisation (n = 1223 (51%) vs. n = 1177(49%)) and emergency versus elective hospitalisation (n = 849 (36%) vs. 1531 (64%)). Click here for file

Additional file 4 Correlation of the answers of the patients on both scales - Question on behavioural intent to return. Click here for file

Additional file 5 Correlation of the answers of the patients on both scales - Question regarding quality of medical information by nurses. Click here for file

Additional file 6 Correlation of the answers of the patients on both scales - Question concerning judgement whether treated with respect and dignity. Click here for file

Additional file 7 Subgroup results. Main analysis repeated in an exploratory way across the prespecified subgroups short versus long hospital stay and emergency versus elective admission. Click here for file
