# Supplementary material for: Designing questionnaires: healthcare survey to compare two different response scales
Source: BMC Med Res Methodol. 2014 Aug 3;14:96. doi: 10.1186/1471-2288-14-96 (PMC4126910; doi:10.1186/1471-2288-14-96)
Supplement: Additional file 1 — Questionnaire on labelled response scale, entire version. Entire version of the questionnaire on a labelled adjectival response scale, displayed in the original sequence of questions. Questions number 1, 2, 7, 16 and 17 correspond to the questions 3, 4, 5, 2 and 1 respectively of the national survey. [file 1471-2288-14-96-S1.pdf]

Additional file 1: Questionnaire on a labelled response scale, entire version

Entire version of the local part of the questionnaire displayed in the original sequence of questions. Questions number 1, 2, 7, 16 and 17 correspond to the questions 3, 4, 5, 2 and 1 respectively of the national part of the questionnaire.

Your opinion is important for us!

1. When you had important questions for a doctor, did you get answers you could understand?

 Yes, always Yes, sometimes No I didn't have any questions

2. When you had important questions for a nurse, did you get answers you could understand?

 Yes, always Yes, sometimes No I didn't have any questions

3. Sometimes in the hospital, one doctor or nurse will say one thing and another will say something quite different. Did this happen to you?

 Yes, often Yes, sometimes No

4. If you had any anxieties or fears regarding your condition or treatment, did a doctor discuss them with you?

 Yes, completely Yes, somewhat No I didn't have any anxiety nor fears

5. Did the doctors talk in front of you as if you weren't there?

 Yes, often Yes, sometimes No

6. Did you have enough say in your treatment?

 Yes, definitively Yes, sometimes No

7. Did you feel like you were treated with respect and dignity while you were in the hospital?

 Yes, always Yes, sometimes No

8. If you had any anxieties or fears about your condition or treatment, did a nurse discuss them with you?

 Yes, completely Yes, somewhat No I didn’t have any anxiety nor fears

9. Was it easy for you to find someone on the hospital staff to talk to about your concerns?

 Yes, definitively Yes, somewhat No I didn’t have any concerns

10a. Were you ever in any pain?

 Yes No

10b. If yes: Do you think that the hospital staff did everything they could to help control the pain?

 Yes, sure Yes, somewhat No

11. Did your family or someone else close to you have enough opportunity to talk to your doctor?

 Yes, definitively Yes, somewhat No There were no family or friends involved

 The family didn’t want or need to talk

12. Did the doctors and nurses give your family (or someone close to you) all the information they needed to help you to recover?

 Yes, definitively Yes, somewhat No There were no family or friends involved

 The family didn’t want or need information

13. Did one of the hospital staff explain the purpose of the medication you were to take at home, in a way you could understand?

 Yes, definitively Yes, somewhat No I needed no explanation

 I had no medication to take at home

14. Did someone tell you about the medication side effects to watch out for after you went home?

 Yes, completely Yes, somewhat No I needed no explanation

 I had no medication to take at home

15. Did they tell you about the dangerous symptoms associated with your illness or operation to watch for after you went home?

 Yes, completely Yes, somewhat No

16. How do you rate the quality of treatment you were given?

 Excellent Good Fair Poor

17. Would you come again to give birth or to be treated for a disease in our hospital?

 Yes, of course Yes, I think so No, I don’t think so Of course not
