# Supplementary material for: Designing questionnaires: healthcare survey to compare two different response scales
Source: BMC Med Res Methodol. 2014 Aug 3;14:96. doi: 10.1186/1471-2288-14-96 (PMC4126910; doi:10.1186/1471-2288-14-96)
Supplement: Additional file 2 — Baseline characteristics of patients without returned questionnaire. Characteristics of patients who did not their questionnaire: All patients (n = 4201 (59%)) and within the pre-defined subgroups short versus long hospitalisation (n = 2243 (53%) vs. n = 1958 (47%)) and emergency versus elective hospitalisation (n = 2040 (49%) vs. n = 2122 (51%)). [file 1471-2288-14-96-S2.pdf]

Additional file 2: Baseline characteristics of patients without returned questionnaire.

Characteristics of patients who did not return their questionnaire: all patients (n=4201 (59%)) and within the pre-defined subgroups short versus long hospital stay (n=2243 (53%) vs. n=1958 (47%)) and emergency versus elective admission (n=2040 (49%) vs. n=2122 (51%))

		Length of Hospital Stay (n=4201)		Admission (n=4162)	
	Total (n=4201)	Length of Stay ≤ 4 Days n=2243 (53%)	Length of Stay > 4 Days n=1958 (47%)	Emergency Admission n=2040 (49%)	Elective Admission n=2122 (51%)
**Age in years**, mean (SD)	58 (20)	55 (21)	62 (19)	63 (21)	54 (19)
**Gender**, n (%)					
Male	2004 (48%)	1072 (48%)	932 (48%)	1013 (50%)	967 (46%)
Female	2197 (52%)	1171 (52%)	1026 (52%)	1027 (50%)	1155 (54%)
**Length of hospital stay**, median (IQR)	4 (2 – 9)	2 (1 – 3)	9 (7 – 14)	5 (1 – 10)	4 (2 - 7)
**Hospitalisation,** n (%)					
Emergency	2040 (49%)	991 (44%)	1049 (54%)		
Elective	2122 (51%)	1236 (55%)	886 (45%)		
Not Defined	39 (1%)	16 (1%)	23 (1%)		
Length of Stay ≤ 4 Days	2243 (53%)			991 (49%)	1236 (58%)
Length of Stay > 4 Days	1958 (47%)			1049 (51%)	886 (42%)
**Department**, n (%)					
Surgery	1516 (36%)	687 (31%)	829 (42%)	713 (35%)	771 (36%)
Internal Medicine	1567 (37%)	838 (37%)	729 (37%)	1015 (50%)	552 (26%)
Gynaecology and Obstetrics	514 (12%)	321 (14%)	193 (10%)	53 (3%)	461 (22%)
Otorhinolaryngology	172 (4%)	132 (6%)	40 (2%)	50 (2%)	118 (6%)
Radiology	85 (2%)	84 (4%)	1 (<1%)	22 (1%)	85 (4%)
Ophthalmology	82 (2%)	67 (3%)	15 (1%)	103 (5%)	59 (3%)
Geriatric Medicine	114 (3%)	9 (<1%)	105 (5%)	78 (4%)	11 (1%)
Intensive Care Unit	115 (3%)	95 (4%)	20 (1%)	5 (<1%)	35 (2%)
Dermatology and Venereology	35 (1%)	9 (<1%)	26 (1%)	52 (5%)	30 (1%)
Missing	1 (<1%)	1 (<1%)		1 (<1%)	

	Total	Length of Hospital Stay (n=4201)		Admission (n=4162)	
		Length of Stay ≤ 4 Days	Length of Stay > 4 Days	Emergency Admission	Elective Admission
	(n=4201)	n=2243 (53%)	n=1958 (47%)	n=2040 (49%)	n=2122 (51%)
**Nationality,** n (%)					
Swiss	2899 (69%)	1446 (64%)	1453 (74%)	1490 (73%)	1383 (65%)
German, Austrian, Liechtensteiner	312 (7%)	191 (9%)	121 (6%)	152 (7%)	158 (7%)
French	48 (1%)	23 (1%)	25 (1%)	19 (1%)	27 (1%)
Italian	175 (4%)	95 (4%)	80 (4%)	101 (5%)	74 (3%)
English, Irish	38 (1%)	22 (1%)	16 (1%)	16 (1%)	21 (1%)
Turkish	143 (3%)	91 (4%)	52 (3%)	53 (3%)	88 (4%)
European, other	297 (7%)	182 (8%)	115 (6%)	115 (6%)	179 (8%)
US-American, Canadian, Australian	36 (1%)	24 (1%)	12 (1%)	12 (1%)	23 (1%)
Extra-European, other	202 (5%)	132 (6%)	70 (4%)	73 (4%)	129 (6%)
Missing	51 (1%)	37 (2%)	14 (1%)	9 (<1%)	40 (2%)
**Language**, n (%)					
German	3971 (95%)	2101 (94%)	1870 (96%)	1938 (95%)	1996 (94%)
French	39 (1%)	22 (1%)	17 (1%)	16 (1%)	22 (1%)
Italian	44 (1%)	22 (1%)	22 (1%)	30 (1%)	14 (1%)
English	57 (1%)	42 (2%)	15 (1%)	19 (1%)	37 (2%)
Turkish	47 (1%)	28 (1%)	19 (1%)	23 (1%)	24 (1%)
Others	43 (1%)	28 (1%)	15 (1%)	14 (1%)	29 (1%)
