# Supplementary material for: Designing questionnaires: healthcare survey to compare two different response scales
Source: BMC Med Res Methodol. 2014 Aug 3;14:96. doi: 10.1186/1471-2288-14-96 (PMC4126910; doi:10.1186/1471-2288-14-96)
Supplement: Additional file 3 — Baseline characteristics of patients with entire completion of all questions on both scales. Characteristics of patients who returned the questionnaire and completely filled out all questions on both response scales corresponding to the analysis population for response scale comparison: All patients (n = 2400 (34%)) and within the pre-defined subgroups short versus long hospitalisation (n = 1223 (51%) vs. n = 1177(49%)) and emergency versus elective hospitalisation (n = 849 (36%) vs. 1531 (64%)). [file 1471-2288-14-96-S3.pdf]

Additional file 3: Baseline characteristics of patients with entire completion of all questions on both scales.

Characteristics of patients who returned the questionnaire and completely filled out all questions on both response scales corresponding to the analysis population for response scale comparison: all patients (n= 2400 (34%)) and within the pre-defined subgroups short versus long hospital stay (n=1223 (51%) vs. n=1177 (49%)) and emergency versus elective admission (n=849 (36%) vs. n=1531 (64%))

		Length of Hospital Stay (n=2400)		Admission (n=2380)	
	Total (n=2400)	Length of Stay ≤ 4 Days n=1223 (51%)	Length of Stay > 4 Days n=1177 (49%)	Emergency Admission n=849 (36%)	Elective Admission n=1531 (64%)
**Age in years**, mean (SD)	59 (19)	56 (19)	62 (18)	63 (19)	56 (18)
**Gender**, n (%)					
Male	1191 (50%)	701 (47%)	718 (49%)	468 (55%)	709 (46%)
Female	1209 (50%)	781 (53%)	757 (51%)	381 (45%)	822 (54%)
**Length of hospital stay**, median (IQR)	4 (2 – 9)	2 (1 – 3)	9 (7 – 13)	6 (2 – 11)	4 (2 – 8)
**Hospitalisation,** n (%)					
Emergency	849 (35%)	350 (29%)	499 (42%)		
Elective	1531 (64%)	869 (71%)	662 (56%)		
Not Defined	20 (1%)	4 (<1%)	16 (1%)		
Length of Stay ≤ 4 Days	1223 (51%)			350 (41%)	869 (57%)
Length of Stay > 4 Days	1177 (49%)			499 (59%)	662 (43%)
**Department**, n (%)					
Surgery	959 (40%)	349 (29%)	610 (52%)	332 (39%)	609 (40%)
Internal Medicine	754 (31%)	408 (33%)	346 (29%)	402 (47%)	352 (23%)
Gynaecology and Obstetrics	371 (15%)	232 (19%)	139 (12%)	19 (2%)	351 (23%)
Otorhinolaryngology	95 (4%)	79 (6%)	16 (1%)	13 (2%)	81 (5%)
Radiology	65 (3%)	65 (5%)	0 (0%)	0 (0%)	65 (4%)
Ophthalmology	46 (2%)	43 (4%)	3 (<1%)	11 (1%)	35 (2%)
Geriatric Medicine	43 (2%)	2 (<1%)	41 (3%)	39 (5%)	4 (<1%)
Intensive Care Unit	41 (2%)	39 (3%)	2 (<1%)	27 (3%)	14 (1%)
Dermatology and Venereology	26 (1%)	6 (<1%)	20 (2%)	6 (1%)	20 (1%)

	Total	Length of Hospital Stay (n=2400)		Admission (n=2380)	
	Total (n=2400)	Length of Stay ≤ 4 Days n=1223 (51%)	Length of Stay > 4 Days n=1177 (49%)	Emergency Admission n=849 (36%)	Elective Admission n=1531 (64%)
**Nationality,** n (%)					
Swiss	1815 (76%)	877 (72%)	938 (80%)	675 (80%)	1126 (74%)
German, Austrian, Liechtensteiner	189 (8%)	108 (9%)	81 (7%)	55 (6%)	132 (9%)
French	30 (1%)	19 (2%)	11 (1%)	4 (<1%)	26 (2%)
Italian	84 (4%)	42 (3%)	42 (4%)	35 (4%)	48 (3%)
English, Irish	9 (<1%)	4 (<1%)	5 (<1%)	6 (1%)	3 (<1%)
Turkish	43 (2%)	26 (2%)	17 (1%)	12 (1%)	30 (2%)
European, other	132 (6%)	82 (7%)	50 (4%)	39 (5%)	93 (6%)
US-American, Canadian, Australian	15 (1%)	14 (1%)	1 (<1%)	4 (<1%)	10 (1%)
Extra-European, other	50 (2%)	30 (2%)	20 (2%)	17 (2%)	33 (2%)
Missing	33 (1%)	21 (2%)	12 (1%)	2 (<1%)	30 (2%)
**Language**, n (%)					
German	2267 (94%)	1147 (94%)	1120 (95%)	808 (95%)	1442 (94%)
French	39 (2%)	22 (2%)	17 (1%)	8 (1%)	31 (2%)
Italian	33 (1%)	16 (1%)	17 (1%)	17 (2%)	14 (1%)
English	24 (1%)	18 (1%)	6 (1%)	9 (1%)	15 (1%)
Turkish	13 (1%)	6 (<1%)	7 (1%)	2 (<1%)	10 (1%)
Others	24 (1%)	14 (1%)	10 (1%)	5 (1%)	19 (1%)
