# Supplementary figures and images for: Designing questionnaires: healthcare survey to compare two different response scales
Source: BMC Med Res Methodol. 2014 Aug 3;14:96. doi: 10.1186/1471-2288-14-96 (PMC4126910; doi:10.1186/1471-2288-14-96)

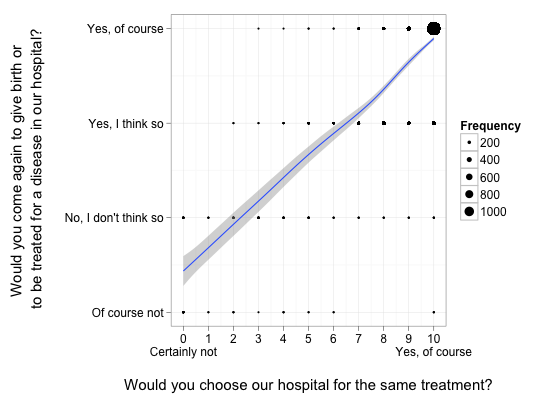

Supplement: Additional file 4 — Correlation of the answers of the patients on both scales - Question on behavioural intent to return. [file 1471-2288-14-96-S4.png]

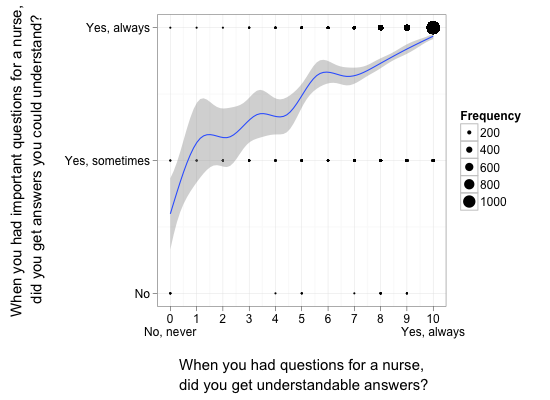

Supplement: Additional file 5 — Correlation of the answers of the patients on both scales - Question regarding quality of medical information by nurses. [file 1471-2288-14-96-S5.png]

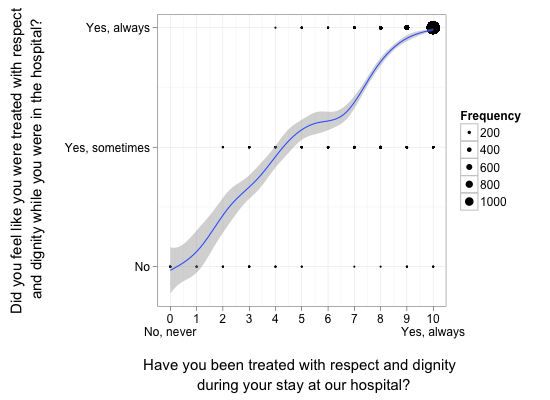

Supplement: Additional file 6 — Correlation of the answers of the patients on both scales - Question concerning judgement whether treated with respect and dignity. [file 1471-2288-14-96-S6.png]
