# Supplementary material for: Designing questionnaires: healthcare survey to compare two different response scales
Source: BMC Med Res Methodol. 2014 Aug 3;14:96. doi: 10.1186/1471-2288-14-96 (PMC4126910; doi:10.1186/1471-2288-14-96)
Supplement: Additional file 7 — Subgroup results. Main analysis repeated in an exploratory way across the prespecified subgroups short versus long hospital stay and emergency versus elective admission. [file 1471-2288-14-96-S7.pdf]

Additional file 5: Subgroup results

Main analysis repeated in an exploratory way across the prespecified subgroups short versus long hospital stay and emergency versus elective admission.

NS=Numeric Scale, LS=Labelled Scale. LL=Lower Limit. CI=Confidence Interval.

NA = I didn't have any questions for staff.

Corr. Coeff. Correlation Coefficient

a) Questionnaire results on a numeric or a labelled adjectival response scale (n = 2400)

The answering scale in the NS had anchoring labels at 0 and 10 of

- 'Certainly not – Yes, of course' for the first question,

- 'Poor – Excellent' for the second question, and

- 'No, never – Yes, always' for questions 3 to 5, respectively.

The categories of the answering scale in the LS were labelled with

- 'Of course not (A) – No, I don't think so (B) – Yes, I think so (C) – Yes, of course (D)' for the first question,

- 'Poor (A) – Fair (B) – Good (C) – Excellent (D)' for the second question, and

- 'No (A) – Yes, sometimes (B) – Yes, always (C) –for questions 3 to 5, respectively.

According to length of hospital stay (n=2400)

		Numeric Scale (NS)												Labelled Scale (LS)				
Questions	Subgroup	0	1	2	3	4	5	6	7	8	9	10	NA	A	B	C	D	NA
Return	≤ 4 days<br>> 4 days	1%<br>1%	<1%<br><1%	1%<br>1%	<1%<br>1%	1%<br>1%	1%<br>2%	1%<br>1%	4%<br>4%	11%<br>11%	12%<br>14%	67%<br>66%	-	1%<br>1%	3%<br>2%	21%<br>25%	75%<br>72%	-
Quality	≤ 4 days<br>> 4 days	<1%<br><1%	<1%<br><1%	<1%<br><1%	1%<br>1%	1%<br>1%	1%<br>2%	2%<br>2%	5%<br>5%	15%<br>15%	23%<br>22%	52%<br>52%	-	1%<br>1%	3%<br>3%	36%<br>41%	60%<br>55%	-
Question to Physicians	≤ 4 days<br>> 4 days	<1%<br><1%	<1%<br><1%	<1%<br><1%	1%<br>1%	1%<br>1%	2%<br>2%	1%<br>3%	4%<br>5%	13%<br>15%	15%<br>15%	56%<br>53%	7%<br>4%	<1%<br>1%	13%<br>17%	77%<br>76%	-	10%<br>6%
Question to Nurses	≤ 4 days<br>> 4 days	<1%<br><1%	<1%<br><1%	<1%<br><1%	<1%<br>1%	<1%<br>1%	3%<br>2%	2%<br>4%	5%<br>6%	16%<br>15%	16%<br>18%	48%<br>47%	8%<br>6%	1%<br>1%	13%<br>16%	81%<br>80%	-	5%<br>4%
Respect and Dignity	≤ 4 days<br>> 4 days	<1%<br><1%	<1%<br><1%	<1%<br><1%	1%<br>1%	1%<br>1%	1%<br>1%	1%<br>1%	3%<br>3%	8%<br>9%	15%<br>15%	71%<br>68%	-	1%<br>2%	8%<br>10%	91%<br>89%	-	-

According to hospital admission type (n=2380, i.e. 20 missing values in admission type)

		Numeric Scale (NS)												Labelled Scale (LS)				
Questions	Subgroup	0	1	2	3	4	5	6	7	8	9	10	NA	A	B	C	D	NA
Return	Emergency<br>Elective	1%<br>1%	<1%<br><1%	1%<br>1%	1%<br><1%	1%<br><1%	2%<br>1%	1%<br>1%	5%<br>4%	11%<br>11%	13%<br>12%	63%<br>69%	-	1%<br>1%	3%<br>2%	28%<br>20%	67%<br>76%	-
Quality	Emergency<br>Elective	<1%<br><1%	-<br><1%	<1%<br><1%	1%<br><1%	1%<br>1%	2%<br>1%	3%<br>1%	6%<br>4%	16%<br>15%	20%<br>24%	51%<br>53%	-	1%<br>1%	4%<br>3%	45%<br>35%	51%<br>61%	-
Question to Physicians	Emergency<br>Elective	<1%<br><1%	<1%<br><1%	<1%<br><1%	1%<br><1%	1%<br>1%	3%<br>2%	3%<br>2%	5%<br>4%	15%<br>13%	14%<br>16%	49%<br>58%	8%<br>4%	1%<br><1%	16%<br>14%	73%<br>79%	-	10%<br>7%
Question to Nurses	Emergency<br>Elective	<1%<br><1%	<1%<br><1%	<1%<br><1%	1%<br><1%	1%<br>1%	2%<br>2%	2%<br>2%	7%<br>5%	16%<br>16%	16%<br>18%	45%<br>49%	9%<br>6%	1%<br>1%	17%<br>13%	75%<br>83%	-	7%<br>3%
Respect and Dignity	Emergency<br>Elective	<1%<br><1%	<1%<br><1%	<1%<br><1%	1%<br><1%	1%<br>1%	1%<br>1%	1%<br>1%	4%<br>2%	10%<br>8%	14%<br>16%	66%<br>71%	-	2%<br>1%	10%<br>8%	88%<br>91%	-	-

b) Spearman's rank correlation coefficient between NS and LS for each item

Spearman's rank correlation coefficient for each item between both response scales (NS and LS): all patients (n=2400 for question 1, 2, and 5, n=2140 for question 3 and n=2173 for question 4, respectively) and for pre-defined subgroups short versus long hospital stay and emergency versus elective admission

	Hospital Stay ≤ 4 days (n=1223)		Hospital Stay > 4 days (n=1177)	
Question	Corr. Coeff.	95% CI	Corr. Coeff.	95% CI
Return	0.61	0.58, 0.65	0.60	0.56, 0.63
Quality	0.63	0.60, 0.67	0.54	0.50, 0.58
Question to Doctors	0.27	0.21, 0.32	0.37	0.32, 0.42
Question to Nurses	0.34	0.28, 0.39	0.33	0.27, 0.38
Respect and Dignity	0.52	0.48, 0.56	0.51	0.47, 0.55
	Emergency Admission (n=849)		Elective Admission (n=1531)	
Question	Corr. Coeff.	95% CI	Corr. Coeff.	95% CI
Return	0.61	0.57, 0.65	0.60	0.56, 0.63
Quality	0.59	0.54, 0.63	0.59	0.56, 0.62
Question to Doctors	0.35	0.29, 0.42	0.30	0.25, 0.35
Question to Nurses	0.38	0.31, 0.44	0.31	0.26, 0.35
Respect and Dignity	0.55	0.51, 0.60	0.50	0.46, 0.53

c) Cronbach's alpha for both response scales

	Hospital Stay ≤ 4 days (n=1223)		Hospital Stay > 4 days (n=1177)	
Scale	Cronbach's alpha	LL of 95% CI	Cronbach's alpha	LL of 95% CI
NS	0.89	0.883	0.89	0.885
LS	0.75	0.732	0.78	0.764
	Emergency Admission (n=849)		Elective Admission (n=1531)	
Scale	Cronbach's alpha	LL of 95% CI	Cronbach's alpha	LL of 95% CI
NS	0.89	0.878	0.89	0.886
LS	0.79	0.766	0.75	0.736

d) Percentage score of the total sum of questionnaire answers

Percentage of total sum of all 5 answers compared to the maximum possible sum on each scale. The maximum possible sum was reduced for those patients who said they had no questions for hospital staff: All patients (n=2400) and within pre-defined subgroups short versus long hospital stay and emergency versus elective admission

Percentage Score	Numeric Scale		Labelled Scale	
	Hospital Stay ≤ 4 days	Hospital Stay > 4 days	Hospital Stay ≤ 4 days	Hospital Stay > 4 days
Median (IQR)	96 (88 – 100)	96 (86 – 100)	100 (88 – 100)	94 (88 – 100)
	Emergency Admission	Elective Admission	Emergency Admission	Elective Admission
Median (IQR)	95 (84 – 100)	96 (88 – 100)	94 (88 – 100)	100 (88 – 100)
